# ZBED6 counteracts high-fat diet-induced glucose intolerance by maintaining beta cell area and reducing excess mitochondrial activation

**DOI:** 10.1007/s00125-021-05517-0

**Published:** 2021-07-22

**Authors:** Xuan Wang, Shady Younis, Jing Cen, Yun Wang, Camilla Krizhanovskii, Leif Andersson, Nils Welsh

**Affiliations:** 1 Science for Life Laboratory, Department of Medical Cell Biology, Uppsala University, Uppsala, Sweden; 2 Science for Life Laboratory, Department of Medical Biochemistry and Microbiology, Uppsala University, Uppsala, Sweden; 3 Division of Immunology and Rheumatology, Stanford University, Stanford, CA USA; 4 Department of Veterinary Integrative Biosciences, Texas A & M University, College Station, TX USA; 5 Department of Animal Breeding and Genetics, Swedish University of Agricultural Sciences, Uppsala, Sweden

**Keywords:** Beta cell proliferation, Glucose intolerance, High-fat diet, IGF2, Insulin release, PPAR-gamma related coactivator 1 protein, Pttg1, ZBED6

## Abstract

**Aims/hypothesis:**

ZBED6 (zinc finger, BED-type containing 6) is known to regulate muscle mass by suppression of *Igf2* gene transcription. In insulin-producing cell lines, ZBED6 maintains proliferative capacity at the expense of differentiation and beta cell function. The aim was to study the impact of *Zbed6* knockout on beta cell function and glucose tolerance in C57BL/6 mice.

**Methods:**

Beta cell area and proliferation were determined in *Zbed6* knockout mice using immunohistochemical analysis. Muscle and fat distribution were assessed using micro-computed tomography. Islet gene expression was assessed by RNA sequencing. Effects of a high-fat diet were analysed by glucose tolerance and insulin tolerance tests. ZBED6 was overexpressed in EndoC-βH1 cells and human islet cells using an adenoviral vector. Beta cell cell-cycle analysis, insulin release and mitochondrial function were studied in vitro using propidium iodide staining and flow cytometry, ELISA, the Seahorse technique, and the fluorescent probes JC-1 and MitoSox.

**Results:**

Islets from *Zbed6* knockout mice showed lowered expression of the cell cycle gene *Pttg1*, decreased beta cell proliferation and decreased beta cell area, which occurred independently from ZBED6 effects on *Igf2* gene expression. *Zbed6* knockout mice, but not wild-type mice, developed glucose intolerance when given a high-fat diet. The high-fat diet *Zbed6* knockout islets displayed upregulated expression of oxidative phosphorylation genes and genes associated with beta cell differentiation. In vitro*,* ZBED6 overexpression resulted in increased EndoC-βH1 cell proliferation and a reduced glucose-stimulated insulin release in human islets. ZBED6 also reduced mitochondrial JC-1 J-aggregate formation, mitochondrial oxygen consumption rates (OCR) and mitochondrial reactive oxygen species (ROS) production, both at basal and palmitate + high glucose-stimulated conditions. ZBED6-induced inhibition of OCR was not rescued by IGF2 addition. ZBED6 reduced levels of the mitochondrial regulator PPAR-γ related coactivator 1 protein (PRC) and bound its promoter/enhancer region. Knockdown of PRC resulted in a lowered OCR.

**Conclusions/interpretation:**

It is concluded that ZBED6 is required for normal beta cell replication and also limits excessive beta cell mitochondrial activation in response to an increased functional demand. ZBED6 may act, at least in part, by restricting PRC-mediated mitochondrial activation/ROS production, which may lead to protection against beta cell dysfunction and glucose intolerance in vivo.

**Graphical abstract:**

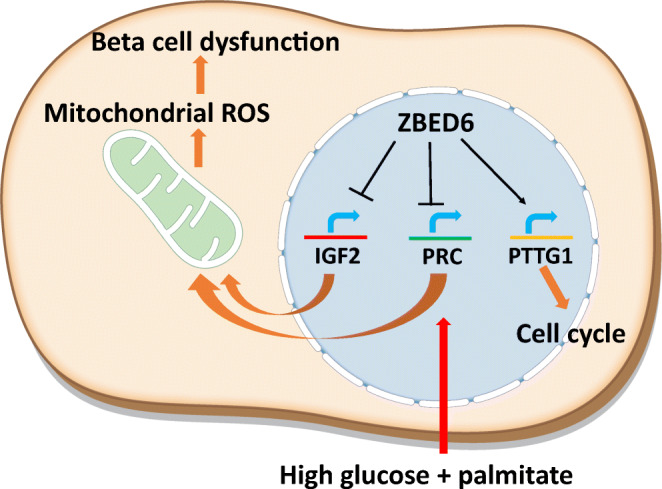

**Supplementary Information:**

The online version contains peer-reviewed but unedited supplementary material available at 10.1007/s00125-021-05517-0.

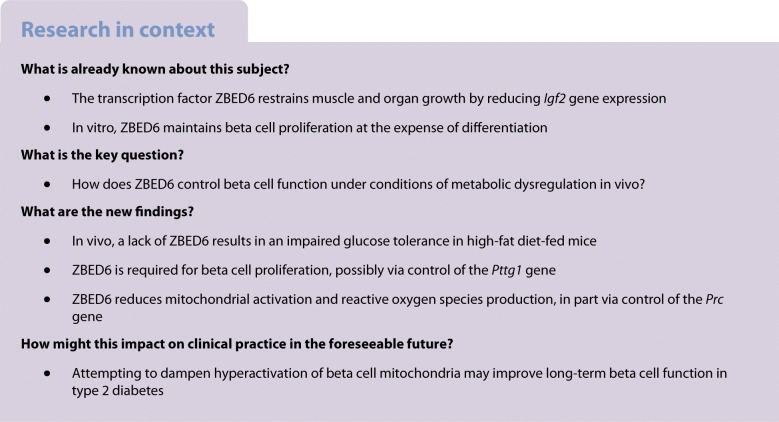



## Introduction

ZBED6 (zinc finger, BED-type containing 6) is a transcriptional modulator that is unique to placental mammals and has evolved from a DNA transposon that integrated in an ancestor of mammals more than 200 million years ago [1]. The fact that the DNA-binding domain of ZBED6 was found to show 100% sequence identity among 25 placental mammals implies that it serves an essential function. ZBED6 acts as a repressor of *Igf2* expression in multiple tissues through the binding of the GCTCG motif located in an intron of *Igf2* [1, 2]. The ZBED6-mediated lowering of *Igf2* expression results not only in decreased muscle mass, but also affects the size of organs such as the liver, kidney and heart [3]. ZBED6 is also expressed in insulin-producing beta cells, where it has been shown to modulate reactive oxygen species (ROS) production, insulin production, proliferation and cell death [4]. In vitro, ZBED6 seems to maintain the capacity of beta cells to proliferate at the expense of specialised function, negatively affecting cell-to-cell interactions [5], stimulus-secretion coupling involving cAMP and Ca^2+^ signalling events, and neuronal/beta cell differentiation pathways [6]. Thus, it is possible that ZBED6 controls the balance between proliferation and function/differentiation of beta cells, thereby preventing either process from unwanted domination. Indeed, in early stages of the pathogenesis of type 2 diabetes, beta cells are often hyperactivated in response to hyperglycaemia, hyperlipidaemia or inflammatory signals [7], leading to hyperinsulinaemia [8]. Later, as peripheral insulin resistance develops and the insulin requirement augments further, the beta cell mass fails to adapt by proliferation [9], and instead de- or transdifferentiate [10], or even undergo apoptosis [11], leading to a worsened glucose homeostasis. This may suggest that initial activation traps the beta cells in a non-proliferative state, which does not allow expansion of the beta cell mass under conditions of continued and intensified stimulation, subsequently causing beta cell damage [12]. Therefore, ZBED6 may be a factor that modulates the balance between proliferation and function. Consequently, in this study we aimed to evaluate the effects of the transcriptional modulator ZBED6 on beta cell proliferation and function in vivo and in vitro.

## Methods

### Animals and high-fat diet treatment

The *Zbed6*^−/−^ and *Igf2*^pA/mG^ models were generated by homologous recombination in mouse *Nnt*^+/+^ C57BL/6 embryonic stem cells as previously described [3], without affecting the host gene *Zc3h11a* [13]. The two transgenic mouse models were both maintained on a C57BL/6 background and bred to generate *Zbed6*^+/+^ *Igf2*^G/G^ (wild-type [WT] G), *Zbed6*^−/−^ *Igf2*^G/G^ (knockout [KO] G), *Zbed6*^+/+^ *Igf2*^pA/mG^ (WT G/A) and *Zbed6*^−/−^ *Igf2*^pA/mG^ (KO G/A) mice. The G to A mutation in the ZBED6 binding site in the *Igf2* intronic CpG island abolishes ZBED6 binding and was present on the paternal chromosome, as *Igf2* is only paternally expressed.

At 5 weeks of age, *Zbed6* KO G and WT G mice (both female and male) were divided into two groups with 16 mice in each. One group was given a control diet (CD) and the other was given a high-fat diet (HFD). The HFD (D12492, Research Diets, USA) contained 60% energy from fat, whereas the CD (D12450B, Research Diets) contained only 10% energy from fat.

### Ethics permission and animal welfare

All animals were group-housed with free access to food and water in the pathogen-free facilities of Uppsala University and Karolinska Institute. All procedures described in this study were approved by the Uppsala Ethical Committee on Animal Research (#C63/15 and #C143/15) and the Stockholm Ethical Committee (#N38/15), following the rules and regulations of the Swedish Animal Welfare Agency, and were in compliance with the European Communities Council Directive of 22 September 2010 (2010/63/EU). All efforts were made to minimise animal suffering and to reduce the number of animals used.

### Blood glucose tolerance test

The mice, after having fasted for approximately 8 h, were given a single i.p. dose of 2.5 g/kg body weight of 30% wt/vol. d-glucose. Blood was drawn from the tail and measured with Freestyle Mini System (Abbott, TheraSense, USA). Blood glucose was determined prior to injection and then at 10, 30, 60 and 120 min after injection.

### Insulin sensitivity test

Mice were given an i.p. injection (1.6 U/kg body weight) of the insulin analogue Actrapid (Novo Nordisk, Bagsværd, Denmark). The animals had free access to food before the insulin injection and were transferred to new cages without food during the measurements.

### Micro-computed tomography

After 10 weeks on a CD or HFD (15–17 weeks of age) the mice were scanned by micro-computed tomography (micro-CT) (Bruker, USA) directly after euthanasia. The scanning protocol was set using voltage 50 kV, current 500 μA and exposure time 60 ms. We scanned at 36 μm with filter Al 0.5 mm, rotation step 0.7, and two connected scans with a total duration time of 3 min 30 s. The amount of abdominal fat was analysed by reconstructing the whole scan in NRecon. In CTAn the volume of interest was determined by the start and end points of the lumber vertebrae. The amount of fat and muscle between vertebrae L6–L4 was analysed.

### Immunohistochemistry

Pancreatic sections, 5 μm thick, were immunostained as previously described [4]. Sections were incubated with primary antibodies (guinea pig anti-human insulin, [1:3000; Reactionlab, USA], anti-glucagon and anti-Ki-67 [1:250, Abcam, UK]) overnight at 4°C. The secondary antibodies (highly cross-adsorbed Alexa Fluor 633 goat anti-guinea pig [1:500; Life Technologies, USA], highly cross-adsorbed Alexa Fluor 555 goat anti-rabbit [1:500; Life Technologies], Alexa Fluor 647 donkey anti-rabbit [1:200, Jackson ImmunoResearch Laboratories, UK]) were added for 1 h. The total pancreas area for each section was measured using ImageJ and a mean for all sections for each mouse, expressed in arbitrary units, was calculated. Beta cell (and alpha cell) mass was semi-quantified similarly and expressed as a ratio to the total pancreas area obtained for the same section. Number of Ki-67-positive beta and alpha cells were counted and expressed as ratio to total number of beta and alpha cells in the same section. At least 30 islets per pancreas, located at three different regions of the pancreas, were analysed.

### Quantitative real-time PCR analysis

Pancreatic islets from WT G and *Zbed6* KO G C57BL/6 mice were prepared by collagenase digestion and cultured overnight as previously described [14]. RNA was purified from islet cells using the Qiagen RNeasy kit (Qiagen, Hilden, Germany), and cDNA was synthesised using avian myeloblastosis virus (AMV) reverse transcriptase (Bio-Rad, Hercules, CA, USA). Real-time q-PCR was performed using the Roche Light Cycler System and the FastStart DNA Master DNA SYBR Green I kit (Roche Diagnostics, Mannheim, Germany). Values were normalised to the relative amounts of GAPDH cDNA. Primer sequences can be found in electronic supplementary material (ESM Table 1).

### Whole transcriptome analysis

Strand-specific mRNA sequencing libraries were generated using the QuantSeq 3′ mRNA-seq library prep kit (Lexogen, Austria). 100 ng of total RNA was poly-A selected using magnetic beads. The libraries were sequenced as 50 bp single reads using an Illumina HiSeq instrument (Illumina, USA). Sequence reads were mapped to the reference mouse genome (mm10) using STAR 2.5.1b. HTSeq-0.6.1 package was used to generate read counts and edgeR package was used to analyse differentially expressed genes. The gene expression was normalised by trimmed mean of M-values method [15] and the abundance of gene expression was calculated as count-per-million reads. For gene ontology analysis, the differentially expressed genes were analysed using the ClusterProfiler R package [16].

### Mitochondrial ROS and membrane potential analysis

Mycoplasma free βTC-6 (RRID: CVCL_0605) and MIN6 (RRID: CVCL_0431) cells were cultured as previously described [4, 6]. Cells were cultivated in 24-well plates (Falcon, USA) to a density about 5 × 10^5^ cells/well. In experiments, cells were treated with sodium palmitate (0.5 mmol/l in 0.5% fatty acid free BSA) + 20 mmol/l glucose. Human EndoC-βH1 (RRID: CVCL_L909) cells were cultured as previously described [17].

Cells were labelled with 1 μmol/l MitoSOX (Thermo Fisher, USA) for 60 min. Cells were then trypsinised and red fluorescence (FL2) was measured using a flow cytometer. For the analysis of mitochondrial membrane potential, cells were labelled with 5 μmol/l of the fluorescent probe JC-1 (Thermo Fisher), which is activated in the mitochondrial matrix when the membrane is hyperpolarised [18], for 30 min. The ratio between the 585 and 530 nm signals was calculated to monitor changes in mitochondrial membrane potential.

### Immunoblotting

Immunoblotting was performed as previously described [4]. The following primary antibodies were used: mouse anti-PRC (1:400; Santa Cruz Biotechnology, USA), rabbit anti-human phospho-PGC1α (S571) (1:200; R & D systems, USA), and mouse anti-α-tubulin (TU-02) (1:500, Santa Cruz). Fluorescent anti-mouse/rabbit secondary antibodies were from Abcam (1:15,000). The bound antibodies were visualised with a LI-COR Odyssey Fc system (LI-COR Biosciences, Lincoln, USA).

### Adenoviral-mediated ZBED6 overexpression

We used an adenoviral vector expressing human ZBED6 protein (untagged) behind the cytomegalovirus (CMV) promoter from Applied Biological Materials (Abm, Canada). An adenoviral vector without any protein expression was used as a control virus. Virus stocks were purified by caesium chloride density-gradient centrifugation (L-80 ultracentrifuge; Beckman Coulter, Fullerton, CA, USA) and semi-quantified by titration. For the adenoviral-mediated transduction, EndoC-βH1 cells were incubated at 37°C for 1 h in a volume of 0.4 ml RPMI-1640 supplemented with 2% FCS and adenoviral vectors at equal concentrations. Two and 3 days after transfection with viral particles at a concentration of 10 and 50 multiplicity of infection (MOI), cells were fixed in ethanol (−20°C) and then stained for 20 min in 5 μg/ml propidium iodide. Cell cycle phases were analysed by flow cytometry.

Adenoviral vectors (10 and 50 MOI) were also used for transduction of human islets. Permission to obtain pancreatic islet tissue from the Nordic Network for Clinical Islet Transplantation was reviewed and approved by the local ethics committee (Regionala etikprövningsnämnden, Uppsala) in Uppsala, Sweden. Human islets were cultured free-floating in Sterilin dishes in CMRL 1066 medium (ICN Biomedicals, Costa Mesa, CA, USA) containing 5.6 mmol/l glucose, 10% FCSand 2 mmol/l l-glutamine for 1–5 days and then dispersed by trypsin treatment for 7–8 min. Dispersed cells were then transduced as given above. Three days after the transduction procedure islet cells were incubated for 60 min in 1.7 mmol/l glucose and an additional 60 min in 17 mmol/l glucose. Insulin release was determined using an ELISA (Mercodia, Sweden).

### Oxygen consumption rates

Oxygen consumption rates (OCR) and extracellular acidification rate (ECAR), which usually represents glycolytic rates, in EndoC-βH1 cells were measured by Extracellular Flux Analyzer XFe96 (Seahorse Bioscience, USA) as previously described [19]. EndoC-βH1 cells were transduced with adenoviral vectors, and after a 48 h culture period, assayed for OCR and ECAR in 5.6 mmol/l glucose, combination of palmitate (1.5 mmol/l with 2% BSA) and high glucose (20 mmol/l), or mouse IGF2 100 ng/ml (R & D Systems) for 1 h.

### Statistical analysis

SigmaStat 4.0 (SYSTAT, USA) was used for all statistical computations. Results are given as means ± SEM. The differences between two groups were determined by Student’s paired *t* test. For multiple comparisons repeated measurements two-way ANOVA followed by Student Newman Keuls post hoc test was used.

## Results

### Knockout of *Zbed6*, but not knockin of *Igf2*, results in reduced beta cell area

**Fig. 1:**
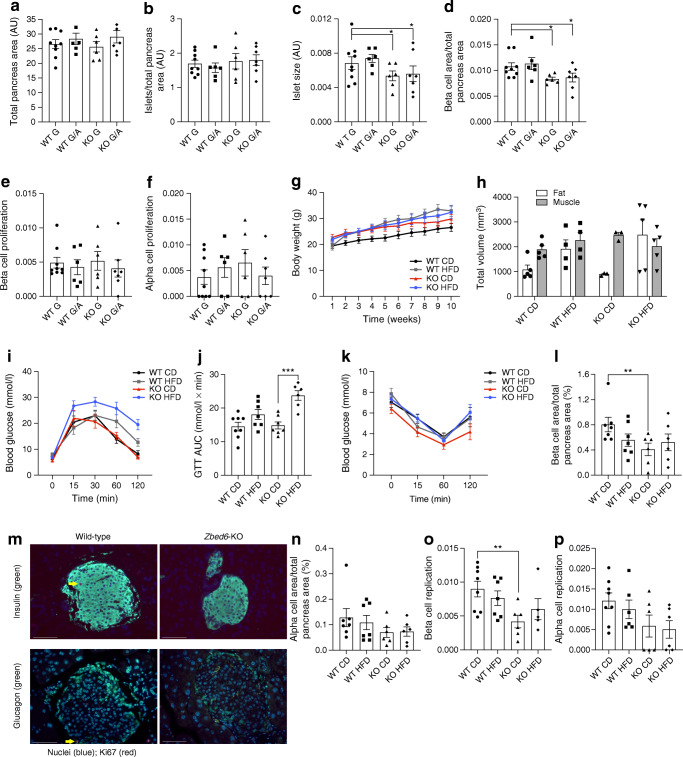
Islet size and beta cell area are reduced in *Zbed6* KO G and *Zbed6* KO G/A mice and *Zbed6* KO promotes HFD-induced glucose intolerance. WT G, KO G, WT G/A and KO G/A mice were killed when 25–28 weeks of age and analysed for (**a**) total pancreas area, (**b**) islet number, (**c**) islet size, (**d**) beta cell area, (**e**) beta cell proliferation and (**f**) alpha cell proliferation. Results are means ± SEM for 6–9 mice per group. *p < 0.05 vs corresponding WT mice. (**g**) Weight curve of WT G and KO G mice given a CD or an HFD from 6 to 15 weeks of age; 7–8 mice per group were analysed. (**h**) Micro-CT was performed on the mice shortly after euthanasia and fat and muscle mass was quantified; 3–5 mice per group were analysed. (**i**) GTT of WT G and KO G mice treated with HFD or CD. Mice were fasted for 8 h and injected intraperitoneally with glucose (2.5 g/kg). Blood glucose levels were analysed at the time points given. Results are means ± SEM for 6–8 mice in each group. (**j**) Data from (**i**) were recalculated to AUC. *** *p* < 0.001 vs KO mice given a CD. (**k**) Insulin sensitivity test of WT G and KO G mice treated for 9   weeks with an HFD. Insulin (1.6 U/kg Actrapid) was injected i.p. and blood glucose was analysed at the time points given in the figure. Results are means ± SEM for 6–8 mice in each group. (**l**) Beta cell area was assessed by morphometric analysis. ** *p* < 0.01 vs WT CD (*n*; = 6–8 mice). (**m**) Representative immunostainings showing morphology of beta cells (insulin, green), alpha cells (glucagon, green) and Ki-67 positive cells (red). Arrows point to two Ki-67 positive beta/alpha cells. White scale bars, 100 μm. (**n**) Alpha cell area was assessed by morphometric analysis (*n* = 6–8 mice). Beta cell (**o**) and alpha cell (**p**) replication was assessed by Ki-67 immunostaining. ** *p* < 0.01 vs WT CD mice (*n* = 6–8 mice)

The *Zbed6* KO mice were generated using the C57BL/6 embryonic stem cell line Bruce4 [20], which are *Nnt*^+^/*Nnt*^+^ as assessed by RNA sequencing (RNA-Seq) analysis of the *Nnt* transcripts (ESM Fig. 1). The general characteristics of the *Zbed6* KO and *Igf2* knockin mice have been recently characterised [3]. To specifically study insulin-producing beta cells, we euthanised *Zbed6*^+/+^ *Igf2*^G/G^ (WT G), *Zbed6*^−/−^ *Igf2*^G/G^ (KO G), *Zbed6*^+/+^ *Igf2*^pA/mG^ (WT G/A) and *Zbed6*^−/−^ *Igf2*^pA/mG^ (KO G/A) mice when 25–28 weeks of age. WT G mice express ZBED6 and low levels of IGF2, the KO G mice lack ZBED6 and express high IGF2 levels, the WT G/A mice do not lack ZBED6 but express high IGF2 levels, and the KO G/A mice lack ZBED6 and express high IGF2 levels [3]. Presently, the total pancreas area and the number of islets per total pancreas area were not affected by ZBED6 or IGF2 (Fig. 1a,b). However, the mean islet size, as well as the beta cell area per total pancreas area, were reduced in both KO G and KO G/A mice (Fig. 1c,d). Thus, the reduced beta cell area occurred in response to *Zbed6* KO and independently from *Igf2* expression. Despite this lowered beta cell area, neither alpha- nor beta cell proliferation were decreased by *Zbed6* KO or *Igf2* knockin (Fig. 1e,f).

### *Zbed6* KO promotes glucose intolerance in HFD-fed mice

Both WT G and KO G mice, 5–7 weeks old, were given a control diet (WT CD and KO CD, respectively) or an HFD (WT HFD and KO HFD, respectively) for 10 weeks. These mice are anticipated to present a mild beta cell phenotype [21]. The HFD increased the weight of WT G mice, but not of KO G mice (Fig. 1g). The weight of KO G mice was higher than that of WT G mice at start of the HFD, which is in line with a previous report stating that muscle and internal organ mass is increased in KO mice [3]. Indeed, using micro-CT we observed an augmented muscle mass of KO CD as compared with WT CD at the level corresponding to the lumbar vertebrae 4 to 6 (L4-L6) (Fig. 1h). In HFD mice, however, this effect was abolished (Fig. 1h).

As in a previous study [3], a GTT showed that KO CD mice responded similarly to the glucose injection as WT CD mice (Fig. 1i). The GTT of WT CD and WT HFD mice were similar. The glucose levels of KO HFD mice were, however, augmented when compared with WT HFD mice (Fig. 1i). Furthermore, calculations of the AUC showed that KO worsened the glucose tolerance in HFD mice (Fig. 1j). We also performed an ITT and observed no differences between the different groups (Fig. 1k), suggesting that neither the 10-week HFD, nor the *Zbed6* KO, affected peripheral insulin sensitivity.

The beta cell area was decreased in KO CD mice as compared with WT CD mice (Fig. 1l), which is in line with the findings with older KO G mice (25–28 weeks) (Fig. 1d). A representative picture of WT G and KO G islets shows that the morphology and size of islet cells was not affected (Fig. 1m). This was paralleled by a lowered beta cell replication rate in the younger (15–17 weeks) KO CD mice (Fig. 1o). This lowering effect by *Zbed6* KO was not observed in HFD-treated mice. There was a non-significant trend to similar effects of *Zbed6* KO on alpha cells (Fig. 1n and p).

### *Zbed6* KO reduces *Pttg1* and increases levels of mRNAs coding for mitochondrial oxidative phosphorylation proteins when exposed to an HFD

**Fig. 2:**
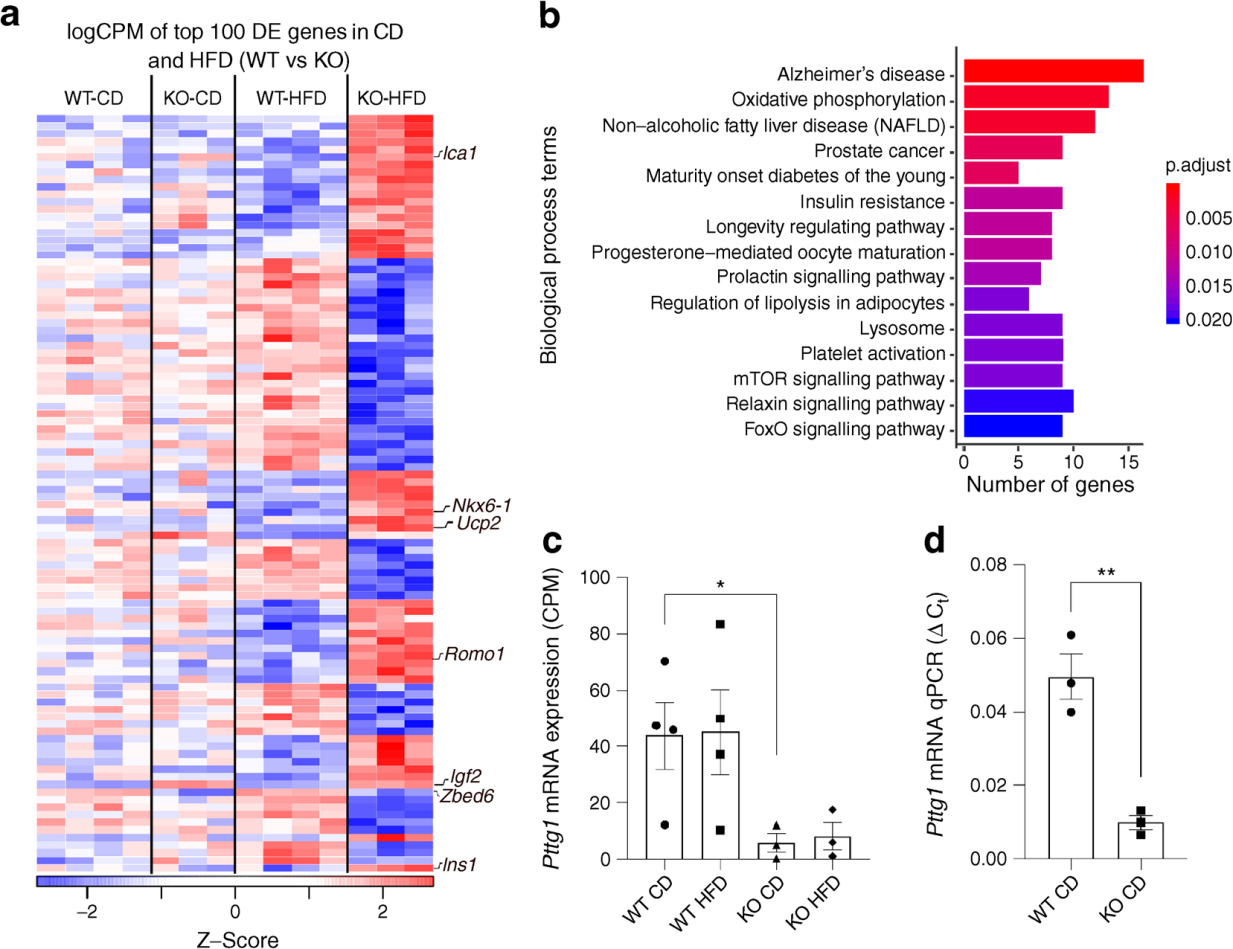
*Zbed6* KO results in decreased islet expression of *Pttg1* and increased expression of oxidative phosphorylation genes when given an HFD. Islets from 3–4 mice in each group were isolated and analysed by RNA-Seq. (**a**) Heat map of the top 100 differentially expressed genes (DEs) in WT HFD vs KO HFD islets, colour scale represents their expression across groups. (**b**) Gene ontology analysis of the upregulated differentially expressed genes in KO HFD. (**c**, **d**) *Pttg1* mRNA expression, as analysed by RNA-Seq (counts per min [CPM]) (**c**) and qPCR (**d**)

RNA-Seq analysis of *Zbed6*-KO G and WT G islets, under CD conditions, revealed up- and downregulation of only six and two genes, respectively (False discovery rate [FDR] < 0.05, Fig. 2a). In contrast, during HFD treatment there was upregulation of 377 genes and downregulation of 409 genes (Fig. 2a, ESM Table 2). Among the downregulated genes we observed that the cell cycle gene *Pttg1* was reduced by almost 90% in the KO islets (ESM Table 2 and Fig. 2c), which was confirmed using RT-qPCR (Fig. 2d). Chromatin immunoprecipitation sequencing (ChIP-seq) using the mouse MIN6 beta cell line indicated a binding site of ZBED6 in the *Pttg1* gene (ESM Fig. 2). In addition to *Pttg1*, we also observed a lowered expression of the cell cycle protein cyclin C (Table 1 and ESM Table 2). Other noteworthy upregulated genes were *Ins1/2*, *Pcsk2* and *Glut2/Slc2a2*, all essential for insulin production (ESM Fig. 3 and Table 1). Beta cell transcription factors, *Nkx6-1*, *FoxO1* and *Pax6* [22], were increased in KO islets (Fig. 2a and Table 1), concurring with previous studies [4, 6]. Also in line with previous reports [5], E-cadherin was downregulated, and N-cadherin and the cAMP-generating enzyme *Adcy9* were upregulated (Table 1). The most important beta cell autoantigens in type 1 diabetes (*Ins1/2*, *Slc30a8*, *Ica1*, *Gad1*, *Ptprn2*) were expressed at higher levels in *Zbed6*-KO islets. Among the upregulated genes in the HFD conditions we observed also *Igf2*, which confirms that ZBED6 acts as a repressor of *Igf2* expression in islet cells (Table 1).

**Table 1:** Genes affected by *Zbed6* knockout in mice. Validations from in vitro beta cell studies were also included

Cellular process	Gene symbol	Gene name	Comment/Reference
Insulin production	*Ins1*	Insulin 1	Upregulated [4, 6]
	*Ins2*	Insulin 2	Upregulated [4, 6]
	*Pcsk2*	Proprotein convertase subtilisin/kexin type 2	Upregulated
	*Slc2a2*	Solute carrier family 2 (facilitated glucose transporter), member 2	Upregulated
Transcription factors	*Nkx6-1*	NK6 homeobox 1	Upregulated [6]
	*Foxo1*	Forkhead box O1	Upregulated
	*Pax6*	Paired box gene 6	Upregulated [6]
Cell cycle	*Pttg1*	Pituitary tumour-transforming gene 1	Downregulated
	*Ccnc*	Cyclin C	Downregulated
Beta cell autoantigens	*Ins1/2*	Insulin 1/Insulin 2	Upregulated
	*Slc30a2*	Solute carrier family 30 (zinc transporter), member 8	Upregulated
	*Ica1*	Islet cell autoantigen 1	Upregulated
	*Gad1*	Glutamic acid decarboxylase 1	Upregulated
	*Ptprn2*	Protein tyrosine phosphatase, receptor type, N polypeptide 2	Upregulated
Cell–cell contacts	*Cdh1*	Cadherin 1 (E-cadherin)	Downregulated [5]
	*Cdh2*	Cadherin 2 (N-cadherin)	Upregulated [5]
cAMP-signalling	*Adcy9*	Adenylate cyclase 9	Upregulated [6]
Mitochondrial ROS	*Romo1*	Reactive oxygen species modulator 1	Upregulated
	*Ucp2*	Uncoupling protein 2 (mitochondrial, proton carrier)	Upregulated
Miscellaneous:	*Igf2*	Insulin-like growth factor 2	Upregulated
	*Zbed6*	Zinc finger, BED-type containing 6	Downregulated

Gene ontology enrichment (GOE) analysis of the KO differentially expressed genes in HFD conditions showed clustering (FDR < 0.05) in the category oxidative phosphorylation among the upregulated genes (Fig. 2b). The 13 genes belonging to this category are shown in ESM Table 3. This was paralleled by increased expression of uncoupling protein 2 (*Ucp2*) and reactive oxygen species modulator 1 (*Romo1*) (Table 1), two proteins involved in mitochondrial ROS production and protection from ROS [23, 24].

### ZBED6 decreases EndoC-βH1 cell basal and maximal OCR

**Fig. 3:**
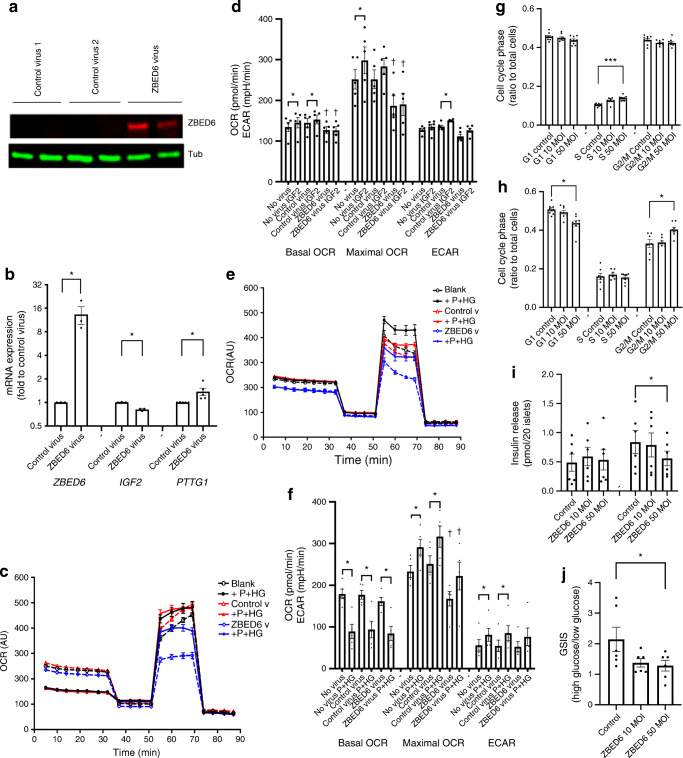
ZBED6 overexpression reduces mitochondrial respiration and glucose-induced insulin release. (**a**) EndoC-βH1 cells were transduced with control (no protein expression) or ZBED6 adenoviral particles and analysed after 2 days by immunoblotting for ZBED6 protein levels. An adenoviral vector without transgene protein expression was used as control virus 1. Control virus 2 was a *SIRT1*-expressing adenoviral vector. The immunoblot is representative for three observations. α-Tubulin (Tub) was used as a control. (**b**) EndoC-βH1 cells were transduced with control (no protein expression) or ZBED6 adenoviral particles and analysed after two days for *ZBED*6, *IGF2* and *PTTG1* mRNA expression levels. Results are normalised to β-actin mRNA and expressed as a ratio to control virus. *p < 0.05 vs control virus for 3–4 independent experiments. (**c**, **d**) Control and ZBED6 overexpressing EndoC-βH1 cells were cultured for 48 h and then pre-incubated at 5.6 mmol/l glucose for 1 h prior to measuring mitochondrial respiration with or without 100 ng/ml IGF2 using the Seahorse technique. Basal OCR, maximal OCR (after FCCP addition) and ECAR were calculated. (**c**) Representative OCR recordings in which each line represents 6–10 replicates, and (**d**) the results from five independent experiments as means ± SEM. *p < 0.05 vs corresponding no IGF2 treatment cells. †*p* < 0.05 vs corresponding control virus cells. (**e**, **f**) Control and ZBED6 overexpressing EndoC-βH1 cells were incubated in the absence and presence of palmitate (1.5 mmol/l in 2% BSA) and high glucose (20 mmol/l) for 1 h during measurement of mitochondrial respiration. Results are mean ± SEM of five independent experiments. *p < 0.05 vs corresponding no treatment cells. † denotes *p* < 0.05 vs corresponding control virus cells. (**g**) EndoC-βH1 cells were transduced with ZBED6 adenoviral vectors at 10 and 50 MOI and then cell cycle phases were analysed by flow cytometry 2 days later. Results are mean ± SEM of five independent experiments. ***p < 0.001 vs corresponding control cells. (**h**) EndoC-βH1 cells were transduced with ZBED6 adenoviral vectors at 10 and 50 MOI and then analysed by flow cytometry 3 days later. Results are mean ± SEM of five independent experiments. *p < 0.05 vs corresponding control cells. (**i**) Human islets were dispersed by trypsin treatment and then transduced with ZBED6 adenoviral vectors at 10 and 50 MOI. Three days later cells were exposed to 1.7 and 17 mmol/l glucose and the insulin release was analysed by ELISA. Results are mean ± SEM of six independent experiments using islets from two human donors. *p < 0.05 vs corresponding control cells. (**j**) Insulin release data from (**i**) were used to calculate the glucose-stimulated insulin release expressed as ratio 17 mmol/l (high) glucose to 1.7 mmol/l (low) glucose. *p < 0.05 vs corresponding control cells

We next determined EndoC-βH1 cell OCR at basal (no addition) and carbonyl cyanide-p-trifluoromethoxyphenylhydrazone (FCCP)-stimulated (maximal OCR) conditions 2 days after ZBED6 adenoviral transduction (Fig. 3a). ZBED6 adenoviral particles increased ZBED6 protein levels in EndoC-βH1 beta cells without signs of cytotoxicity (Fig. 3a, results not shown) and generated an approximate tenfold increase in *ZBED6* mRNA expression (Fig. 3b). ZBED6 overexpression was associated with a lowered *IGF2* mRNA level and an increased *PTTG1* mRNA level (Fig. 3b). ZBED6 overexpression resulted in lower basal and maximal OCR as compared with control virus exposed cells (Fig. 3c,d). IGF2 addition for 1 h stimulated both basal and maximal OCR in control cells, but not in ZBED6 cells. ECAR was unaffected by ZBED6, and was stimulated by IGF2 in control cells, but not in ZBED6 cells (Fig. 3d). Thus, ZBED6 restricts respiration, but not via repression of the *IGF2* gene.

We also analysed effects of ZBED6 overexpression in cells stimulated by palmitate (1.5 mmol/l) and high glucose (20 mmol/l) for 1 h. Addition of palmitate and high glucose increased ECAR and reduced basal OCR (Fig. 3e,f), indicating that EndoC-βH1 cells have a high capacity to upregulate glycolytic ATP production in response to a high glucose concentration (Warburg effect). However, in the presence of FCCP, maximal respiration was increased by palmitate and high glucose in control cells (Fig. 3f), suggesting that palmitate and high glucose can further stimulate respiration at conditions of low mitochondrial ATP. ZBED6 promoted a lowered maximal OCR with and without palmitate and high glucose (Fig. 3f).

ZBED6 overexpression also affected EndoC-βH1 cell proliferation as assessed by analysis of the distribution of cells in the different cell cycle phases. We observed that the fraction of cells in the S phase was increased 2 days after transduction at the concentration of 50 MOI (Fig. 3g). Three days after transfection, the fraction of cells in G1 and G2/M were decreased and increased, respectively, by ZBED6 (Fig. 3h), suggesting that ZBED6 promotes increased proliferation.

To analyse whether ZBED6 affects glucose-induced insulin release we transduced dispersed human islets with ZBED6 adenoviral vectors. After 3 days, the higher concentration of the vector (50 MOI) resulted in a lowered insulin release at 17 mmol/l glucose (Fig. 3i). This resulted in a lowered glucose-stimulated insulin release ratio (Fig. 3j).

### ZBED6 reduces mitochondrial membrane potential and mitochondrial ROS production

**Fig. 4:**
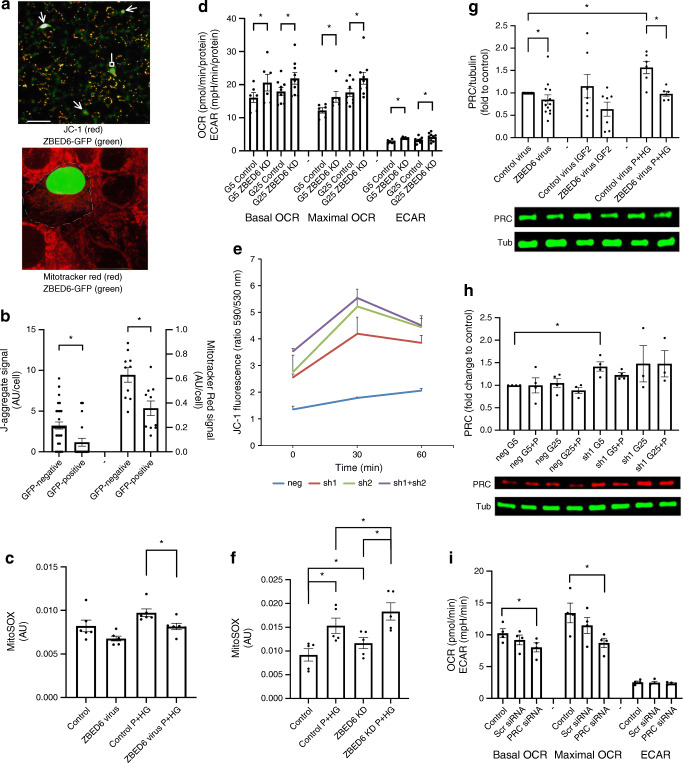
ZBED6 overexpression in EndoC-βH1 cells results in lowered J-aggregate fluorescence, MitoTracker Red uptake and mitochondrial ROS production. Downregulation of ZBED6 results in increased OCR, ECAR, J-aggregate fluorescence and mitochondrial ROS production. ZBED6 overexpression reduces expression level of PRC, and ZBED6 downregulation increases expression level of PRC. (**a**) A fluorescence microscopic image with JC-1 staining (3 μmol/l for 10 min, red colour) of EndoC-βH1 cells showing three ZBED6-GFP-positive cells (green colour, arrows) with no active mitochondria, one ZBED6-GFP-positive cell with active mitochondria (green, block head). Scale bar, 10 μm. Confocal image showing MitoTracker Red staining (10 μmol/l for 10 min, red colour) of a ZBED6-GFP expressing cell (green nucleus) surrounded by non-transfected cells. The dotted line shows the probable cell membrane position of the ZBED6-GFP-positive cell. Scale bar, 50 μm. (**b**) Left axis: quantification of J-aggregate positive mitochondrial structures in GFP-positive and GFP-negative cells. Results are means from 21 (GFP-negative) and 29 (GFP-positive) cells obtained from six randomly photographed culture plate areas. Right axis: quantification of Mitotracker Red signal in GFP-positive and GFP-negative cells. Results are means from ten randomly photographed GFP-positive cells with an adjacent GFP-negative cell. (**c**) MitoSOX fluorescence in EndoC-βH1 control and ZBED6 overexpressing cells quantified by flow cytometry. Cells were cultured for 24 h at standard conditions (5 mmol/l glucose, control) or supplemented with 0.5 mmol/l palmitate + 20 mmol/l glucose (P+HG), and then labelled for 40 min with 1 μmol/l of the MitoSOX probe. Results are means ± SEM for six independent experiments. *p <   0.05. (**d**) Control and *Zbed6* knockdown (ZBED6 KD) MIN6 cells were cultured for 24 h in 5 mmol/l (G5) or 25 mmol/l (G25) glucose and then pre-incubated at 5.6 mmol/l glucose for 1 h prior to measuring mitochondrial respiration at 5 mmol/l (G5) or 25 mmol/l (G25) glucose using the Seahorse technique. Basal OCR, maximal OCR (after FCCP addition) and ECAR were calculated. Results are from six independent experiments. *p < 0.05 vs corresponding control cells. (**e**) βTC-6 cells treated with scrambled shRNA lentiviral particles (negative control; neg) or with two different anti-*Zbed6* shRNA lentiviral particles (sh1 and sh2) were labelled with 3 μmol/l JC-1 for 10 min and then treated with 0.5 mmol/l palmitate +25 mmol/l glucose (P+HG) for 0, 30 or 60 min. J-aggregate fluorescence was obtained by flow cytometry analysis and by calculation of the FL2/FL1 (590/530 nm) ratio. Results are means ± SEM for three experiments. (**f**) MitoSOX fluorescence in MIN6 control and ZBED6 KD cells quantified by flow cytometry. Cells were cultured for 24 h under standard conditions (25 mmol/l glucose, control) or supplemented with 0.5 mmol/l palmitate + 25 mmol/l glucose (P+HG), and then labelled for 40 min at 1 μmol/l MitoSOX probe. Results are means ± SEM for five independent experiments. *p < 0.05. (**g**−**i**) ZBED6 overexpression reduces and ZBED6 downregulation increases expression level of PRC. (**g**) Control and ZBED6 overexpressing EndoC-βH1 cells were cultured for 48 h and then treated with or without mouse IGF2 (100 ng/ml) or the combination palmitate (1.5 mmol/l in 2% BSA) and high glucose (20 mmol/l) for 3 h. The expression level of PRC was measured by immunoblotting analysis. Results are normalised to α-tubulin (Tub) expression level and are expressed as means ± SEM for 6–13 independent experiments. *p <   0.05. (**h**) MIN6 cells expressing scramble shRNA (neg) or anti-*Zbed6* shRNA (sh1) were analysed for PRC expression using immunoblot analysis. Cells were cultured for 24 h in the presence of 5 mmol/l glucose (G5), 5 mmol/l glucose + 0.5 mmol/l palmitate (G5+P), 25 mmol/l glucose (G25) or 25 mmol/l glucose + 0.5 mmol/l palmitate (G25+P). Results are from 3–4 independent experiments. (**i**) Knockdown of PRC results in reduced basal and maximal OCR. EndoC-βH1 cells were transfected with 50 nmol/l siRNA targeting to PRC and then were analysed for OCR using the Seahorse technique. Results are from four independent experiments. *p < 0.05 vs control

We next transfected EndoC-βH1 cells with a ZBED6-GFP expression vector and immunostained for respiratory chain protein ATPase inhibitory factor 1 (ATPIF1), a mitochondrial marker protein, which allows visualisation of mitochondrial density and morphology. ZBED6-GFP-positive cells displayed mitochondria with similar morphology and number as those of non-GFP-positive cells 2 days after transfection (ESM Fig. 4). However, when cells were stained with JC-1, which emits red J-aggregate light when the inner membranes of mitochondria are polarised, GFP-positive cells showed fewer J-aggregate positive mitochondrial structures than GFP-negative cells (Fig. 4a,b). Also, MitoTracker Red uptake into mitochondria of GFP-positive cells was lower than that of GFP-negative cells (Fig. 4a,b). This supports the hypothesis that ZBED6 lowers mitochondrial activity, as MitoTracker Red is not efficiently taken up by inactive mitochondria. Mitochondrial ROS production, assessed using the MitoSOX probe, was lower in ZBED6-overexpressing EndoC-βH1 cells cultured with palmitate and high glucose (Fig. 4c).

### *Zbed6* knockdown results in increased OCR, J-aggregate formation and mitochondrial ROS production

*Zbed6* was knocked down in MIN6 cells by using anti-*Zbed6* short hairpin RNA (shRNA) lentiviral particles [4–6]. As control cells we used scramble shRNA lentiviral-transduced cells (neg) and found that *Zbed6* knockdown (sh1) resulted in higher basal and maximal OCR (Fig. 4d). Also, ECAR was higher in sh1 cells than in neg cells (Fig. 4d). The increased OCR of sh1 cells was observed at both 5 mmol/l and 25 mmol/l glucose.

Silencing of *Zbed6* was also performed in insulin-producing βTC-6 cells [4], and in this case the *Zbed6* knockdown sh1, sh2 and sh1+sh2 cells displayed increased JC-1 J-aggregate fluorescence both at basal conditions and after 30 and 60 min of stimulation with palmitate and high glucose (Fig. 4e).

The MIN6 cells were also used for analysis of mitochondrial ROS production using the MitoSOX probe. The MIN6 sh1 cells produced more mitochondrial ROS, both at basal and palmitate stimulated conditions, than neg cells (Fig. 4f).

### ZBED6 reduces the expression of the mitochondrial regulator PRC

As ZBED6-restricted mitochondrial activity is not restored by exogenous IGF2, we looked for other ZBED6 targets. ZBED6 binds with moderate strength to the PPAR coactivator-1 (PRC) promoter region in MIN6 cells, but very weakly in C2C12 myotube cells [25] (ESM Fig. 2). PRC, together with PGC-1α and PGC-1β, belongs to the PPAR coactivator family and are known activators of mitochondrial protein transcription [26]. In ZBED6-overexpressing EndoC-βH1 cells, PRC levels were lowered at basal conditions and after culture for 24 h in the presence of palmitate and high glucose (Fig. 4g,h). In MIN6 sh1 cells (*Zbed6*-knockdown), PRC was increased at basal conditions (Fig. 4h), suggesting that ZBED6 controls PRC levels in beta cells.

### Knockdown of PRC results in lowered basal and maximal OCR

We next performed RNA interference (RNAi) to downregulate PRC protein levels in EndoC-βH1 cells. In a small interfering RNA (siRNA) titration experiment, we observed after 2 and 3 days an approximate 40–50% lowering of the PRC protein (ESM Fig. 5). In such PRC knockdown cells, basal and maximal OCR were decreased when comparing with scrambled siRNA treatment (Fig. 4i). PRC knockdown did not affect ECAR, suggesting that ZBED6 suppresses mitochondrial function by reducing PRC levels.

## Discussion

ZBED6 is a transcriptional regulator that represses postnatal IGF2 expression [3]. However, we report here an unaltered beta cell area in IGF2 overexpressing WT G/A mice. It is therefore likely that ZBED6-dependent IGF2 expression is without physiological consequence for beta cells, and that the observed ZBED6-induced effects were instead due to other growth-controlling genes. One such gene, which contains ZBED6 binding sites in its promoter/enhancer region and which is downregulated in *Zbed6*-KO mice, is *Pttg1*. The *Pttg1* gene product modulates G1/S cell phase transition [27], functions as a securin during chromosome separation [28] and is required for beta cell development and proliferation [29]. It is therefore possible that ZBED6, by enhancing expression of this and other growth-controlling genes, maintains normal beta cell replication, ensuring an adequate beta cell mass for the long-term preservation of glucose tolerance. Interestingly, ZBED6 expression appears to be reduced in human beta cells with increasing age [30].

The lowered beta cell mass of *Zbed6*-KO mice did not promote glucose intolerance when given an CD, but as the mice were of the *Nnt*^+^/*Nnt*^+^ genotype, a milder beta cell phenotype can be expected [21]. Therefore, it is not surprising that the lowered beta cell mass, without HFD treatment, did not provoke glucose intolerance. However, in *Zbed6*-KO (*Nnt*^+^/*Nnt*^+^) mice fed an HFD, glucose intolerance developed, suggesting that the combination of a reduced beta cell mass and a diet overload is necessary to cause impaired beta cell function.

A clue to why *Zbed6*-KO promotes impaired glucose tolerance in HFD-treated mice may be that the expression of islet oxidative phosphorylation genes was increased in vivo. Interestingly, *Zbed6* knockdown increased and ZBED6 overexpression decreased OCR in vitro, clearly supporting the in vivo results. Mitochondrial respiration is regulated by members of the PPAR coactivator family PRC, PGC-1α and PGC-1β [26]. Human islets express high levels of PRC (6.8 reads per kilobase million [RPKM]), intermediate levels of PGC-1α (2.6 RPKM) and low levels of PGC-1β (0.3 RPKM) [31], and ZBED6 binds to the PRC promoter in beta cells [6]. In line with this, PRC protein levels were increased by *Zbed6*-KO and decreased by ZBED6 overexpression, and PRC knockdown resulted in reduced OCR. This suggests that PRC is an important mitochondrial regulator in human beta cells, and that ZBED6 may control expression of this PPAR coactivator via a direct interaction with the PRC gene promoter/enhancer region. PRC has, to our knowledge, not been investigated in beta cells. Additionally, in other cell types PRC is rather poorly characterised, but it has been proposed that PRC acts rapidly upon extracellular signals, for example growth factor stimulation, to increase mitochondrial biogenesis [26].

There seems to exist a correlation between maximal OCR, inner membrane hyperpolarisation and mitochondrial ROS production, as all three variables were increased by the nutrients palmitate and high glucose. Indeed, it is known that mitochondria of primary beta cells respond to nutrients with stimulation of the Krebs cycle and the respiratory chain [32]. Furthermore, in vitro addition of metabolites, ADP and uncouplers results in superoxide and hydrogen peroxide leakage from the respiratory complexes, and this occurs via chemical reduction of electron leaking sites and a strong protonmotive force [33–35]. Excess nutrients also evoke an increased functional demand (higher need for insulin production), which consumes ATP and increases ADP levels [35], further stimulating the respiratory chain. Moreover, our results demonstrate that mitochondria are activated by the *PRC* gene, a process controlled by ZBED6. Thus, it seems that overnutrition predisposes for augmented beta cell ROS production, and in the case when ZBED6 does not restrict mitochondrial activation by dampening *PRC* gene expression, potentially harmful levels of ROS may be generated. High mitochondrial ROS levels cause beta cell dysfunction [36, 37], and the present ROS increase, in response to nutrients and *Zbed6*-KO, could therefore explain, at least in part, why *Zbed6*-KO mice become glucose intolerant when given an HFD.

Type 2 diabetes is associated with beta cell loss of identity, dedifferentiation or beta to alpha cell transdifferentiation [10, 38], and can in later stages often result in loss of transcription factors such as MafA, Nkx6-1 and Pdx1 [10]. Interestingly, in this study we observed the opposite, namely increased expression of beta cell-specific genes during conditions of glucose intolerance. In addition, important beta cell autoantigens in type 1 diabetes were also increased. Thus, *Zbed6*-KO in functionally challenged mice promoted a beta cell hyper-differentiated and hyper-immunogenic state that mediates not only a higher insulin production, but also might facilitate inflammatory/autoimmune reactions. It is not clear why beta cells of glucose-intolerant *Zbed6*-KO mice become hyper-differentiated rather than dedifferentiated, but it could be envisaged that the islet cells were in an early stage of metabolic stress and still had not started the process of trans-/dedifferentiation [38].

A limitation of this study is that a global knockout was used, making it harder to pinpoint whether ZBED6 affects beta cells directly or via other organs/cell types. Another limitation is that the in vitro techniques were not easily adapted for the in vivo situation. Nevertheless, ZBED6 appears to perform an important function in balancing differentiation vs proliferation. This could promote a more sustainable beta cell phenotype in which mitochondrial ROS production is restrained. The role of ZBED6 in the pathophysiology of type 2 diabetes remains to be clarified, but it is tempting to speculate that ZBED6 maintains beta cell survival and proliferation at the cost of decreased insulin production, and that this is beneficial during long-term high-fat overfeeding.

## Supplementary Information

ESM 1 (PDF 1126 kb)

ESM 2 (XLSX 645 kb)

ESM 3 (XLSX 14 kb)

## Data Availability

The datasets generated during and/or analysed during the current study are available from the corresponding author upon reasonable request.

## Abbreviations

CD: Control diet
CT: Computed tomography
ECAR: Extracellular acidification rate
ES: Embryonic stem
FCCP: Carbonyl cyanide-p-trifluoromethoxyphenylhydrazone
FDR: False discovery rate
HFD: High-fat diet
KO: Knockout
MOI: multiplicity of infection
OCR: Oxygen consumption rate
PRC: PPAR-gamma related coactivator 1 protein
RNA-Seq: RNA sequencing
ROS: Reactive oxygen species
RPKM: Reads per kilobase million
shRNA: Short hairpin RNA
siRNA: Small interfering RNA
WT: Wild-type
ZBED6: Zinc finger, BED-type containing 6
