# Supplementary material for: ZBED6 counteracts high-fat diet-induced glucose intolerance by maintaining beta cell area and reducing excess mitochondrial activation
Source: Diabetologia. 2021 Jul 22;64(10):2292–305. doi: 10.1007/s00125-021-05517-0 (PMC8423654; doi:10.1007/s00125-021-05517-0)
Supplement: Supplementary file 1 — (PDF 1126 kb) [file 125_2021_5517_MOESM1_ESM.pdf]

Checklist for reporting human islet preparations used in research

Adapted from Hart NJ, Powers AC (2018) Progress, challenges, and suggestions for using human islets to understand islet biology and human diabetes. Diabetologia https://doi.org/10.1007/s00125-018-4772-2

Islet preparation	1	2						
**MANDATORY INFORMATION**								
Unique identifier	H2527	H2524						
Donor age (years)	43	69						
Donor sex (M/F)	M	F						
Donor BMI (kg/m^2)	18,9	21,1						
Donor HbA_{1c} or other measure of blood glucose control	HbA_{1c} 33	HbA1c 35						
Origin/source of islets[b]	ECIT	ECIT						
Islet isolation centre	Uppsala	Uppsala						
Donor history of diabetes? Please select yes/no from drop down list	No	No						
**If Yes, complete the next two lines if this information is available**								
Diabetes duration (years)								
Glucose-lowering therapy at time of death[c]								
**RECOMMENDED INFORMATION**								
Donor cause of death								
Warm ischaemia time (h)								
Cold ischaemia time (h)	48h	12:28						
Estimated purity (%)	20	48						
Estimated viability (%)								
Total culture time (h)[d]	All less than 5 days	All less than 5 days						
Glucose-stimulated insulin secretion or other functional measurement[e]		11,4						
Handpicked to purity? Please select yes/no from drop down list	Yes	Yes						

Additional notes								

[a]If you have used more than eight islet preparations, please complete additional forms as necessary
[b]For example, IIDP, ECIT, Alberta IsletCore
[c]Please specify the therapy/therapies
[d]Time of islet culture at the isolation centre, during shipment and at the receiving laboratory
[e]Please specify the test and the results

ESN Table 1. Primer sequences used for qPCR.

**Mouse *Pttg1***	5′-AGTTGCCGAAAAGCCTATGA-3′ (F)
	5′-CCATTCAAGGGGAGAAGTGA-3′ (R)
**Mouse *Gapdh***	5′-AACTTTGGCATTGTGGAAGG-3′ (F)
	5′-GGATGCAGGGATGATGTTCT-3′ (R)
**Human ZBED6**	5′-CAAGACATCTGCCGTTTGGAATTT-3′ (F)
	5′-TGTCGTTGAAGTGTTGAAGTCCCTA-3′ (R)
**Human IGF2**	5′-CGTGGCATCGTTGAGGAG-3′ (F)
	5′-ACACGTCCCTCTCGGACT-3′ (R)
**Human PTTG1**	5′-GAAAATGGAGGACCAGGCAC-3′ (F)
	5′-TCTCAGTCATCTTTTTGGCAG-3′ (R)

ESM Table 2. The list of DE genes in islets of ZBED6 KO and WT mice.
See separate Excel file

ESM Table 3. The GO analysis of up-regulated genes in islets KO vs WT in HFD condition.
See separate Excel File

ESM Fig. 1

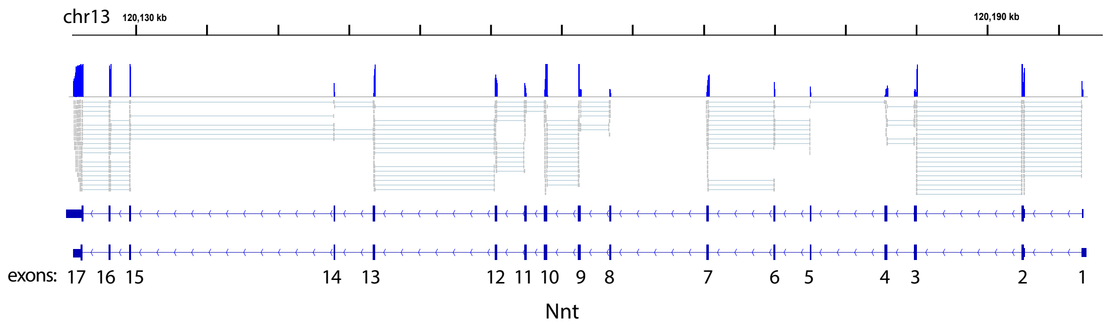

ESM Fig. 1. RNAseq analysis of Nnt gene transcripts.
The Figure shows that all *Nnt* exons are transcribed/expressed in the presently used C57Bl/6 mice, that were derived from the Bruce4 ES cell.

ESM Fig. 2

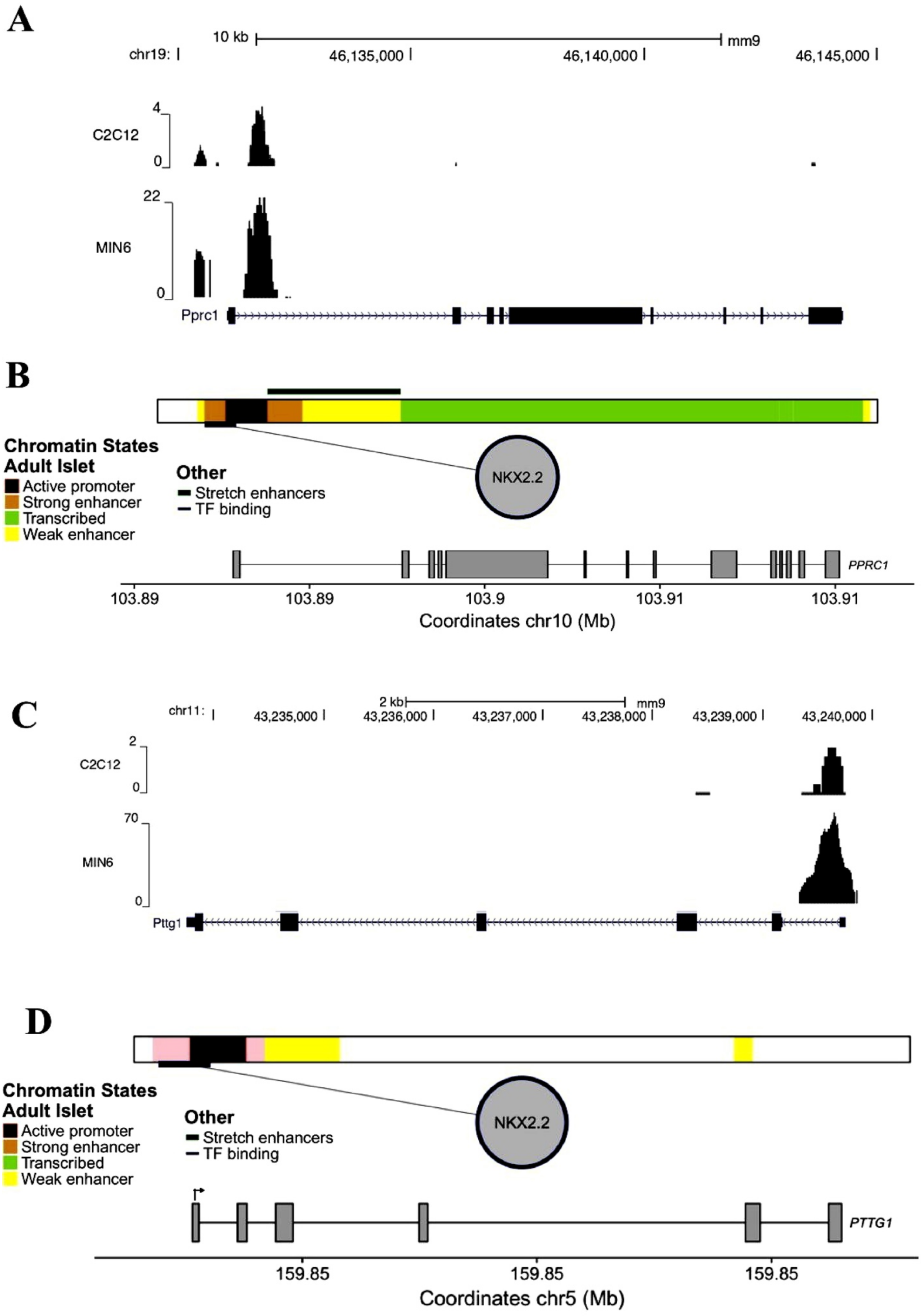
A
10 kb
mm9
chr19:
46,135,000
46,140,000
46,145,000
C2C12
4
0
MIN6
22
0
Pprc1
B
Chromatin States
Adult Islet
Active promoter
Strong enhancer
Transcribed
Weak enhancer
Other
Stretch enhancers
TF binding
NKX2.2
PPRC1
103.89
103.89
103.9
103.91
103.91
Coordinates chr10 (Mb)
C
chr11:
43,235,000
2 kb
mm9
43,236,000
43,237,000
43,238,000
43,239,000
43,240,000
C2C12
2
0
MIN6
70
0
Pttg1
D
Chromatin States
Adult Islet
Active promoter
Strong enhancer
Transcribed
Weak enhancer
Other
Stretch enhancers
TF binding
NKX2.2
PTTG1
159.85
159.85
159.85
Coordinates chr5 (Mb)

ESM Fig. 2. Zbed6 binding sites in the *Prc* and *Pttg1* genes.

ZBED6 binding to the *Prc* gene in MIN6 cells (upper trace) and C2C12 cells (lower trace) as assessed by ChIP analysis. Data are modified from a previous study (13). (B) Depiction of the human *PRC* gene promoter and enhancer regions with sites for beta-cell specific transcription factor binding (NKX2.2) in islet cells (from http://www.isletregulome.org/isletregulome/). The picture has been modified so that the human *PRC* gene is aligned with the mouse *Prc* gene shown in (A). (C) ZBED6 binding to the *Pttg1* gene in MIN6 cells (upper trace) and C2C12 cells (lower trace) (13). (D) Human islet *PTTG1* gene promoter and enhancer regions (from http://www.isletregulome.org/isletregulome/). Observe that the *Pttg1* gene is transcribed from opposite strands in human and mouse.

ESM Fig. 3

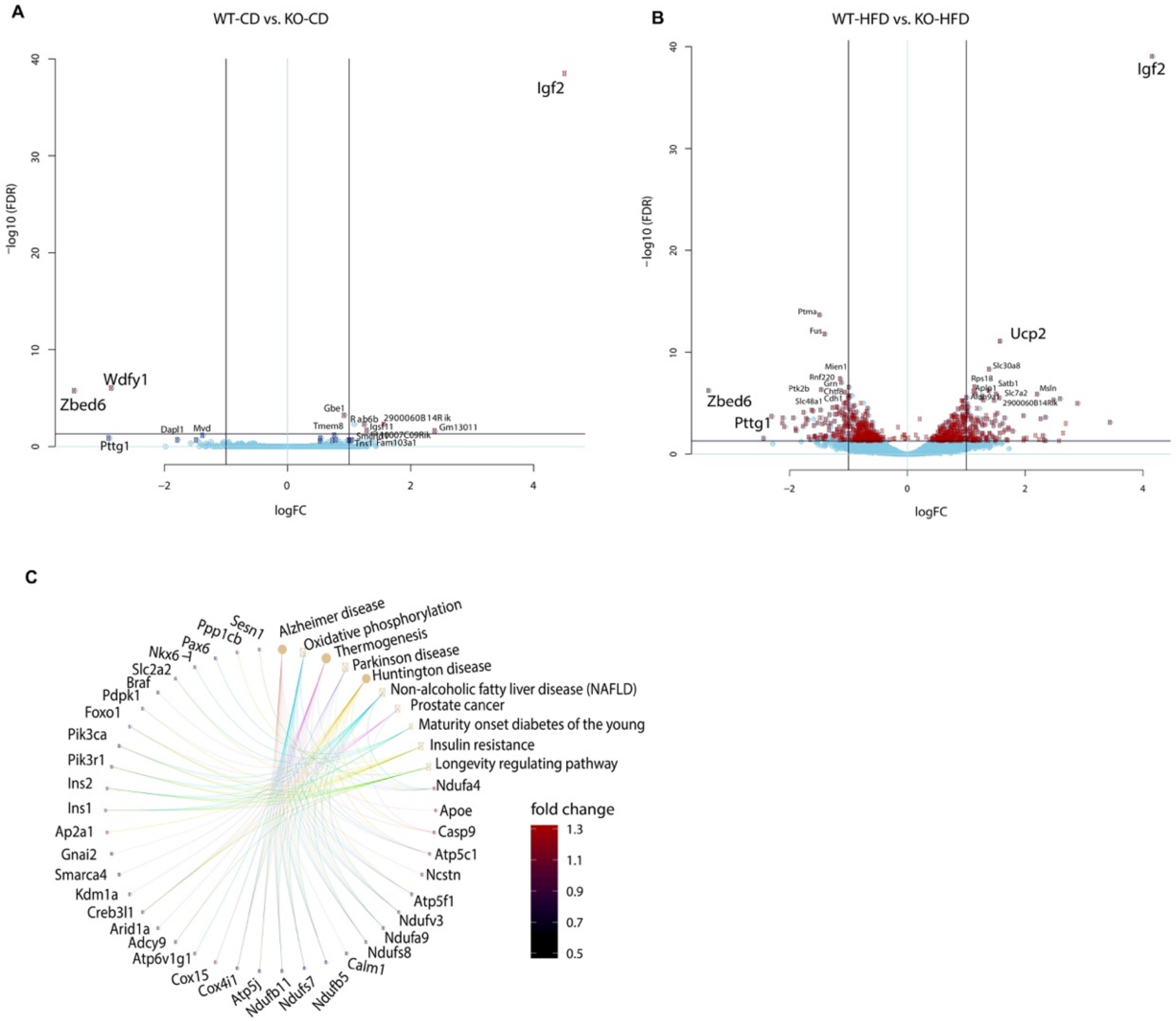

<u>ESM Fig.3. Volcano plot of differentially expressed genes.</u> The values are log-fold changes against -log FDR for the WT vs KO islets in control diet (A) or high fat diet (B). Red dots represent FDR<0.05. (C) Significantly enriched terms in Figure 3B with the name of the genes involved in each term.

ESM Fig. 4

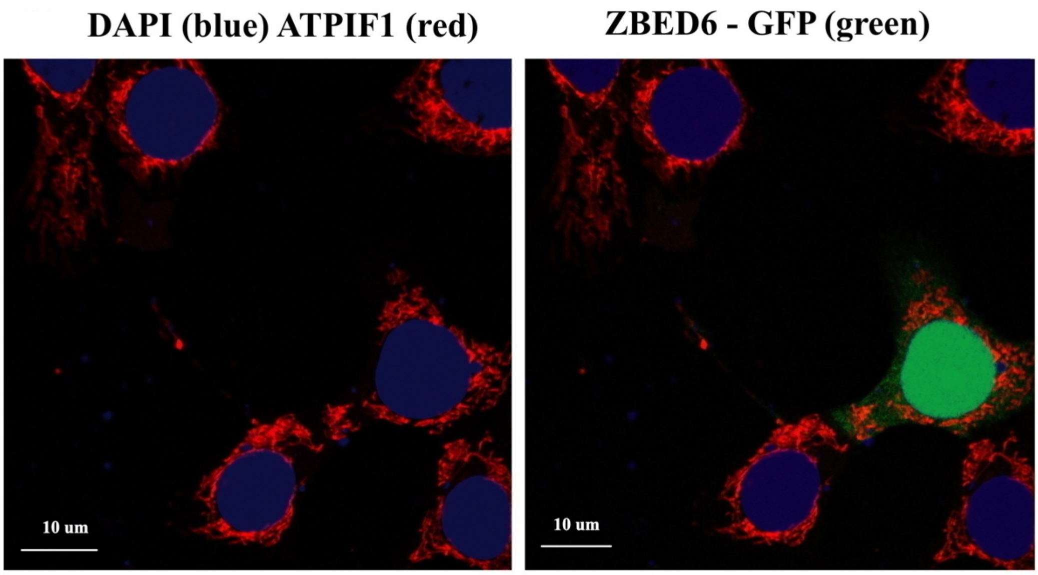

ESM Fig. 4. Immunostaining of EndoC-βH1 cells for the mitochondrial protein ATPIF1 (red). The confocal images show that ZBED6 overexpressing cells, with nuclear GFP-fluorescence, present similar number and size of mitochondria as non-GFP-positive cells.

ESM Fig. 5

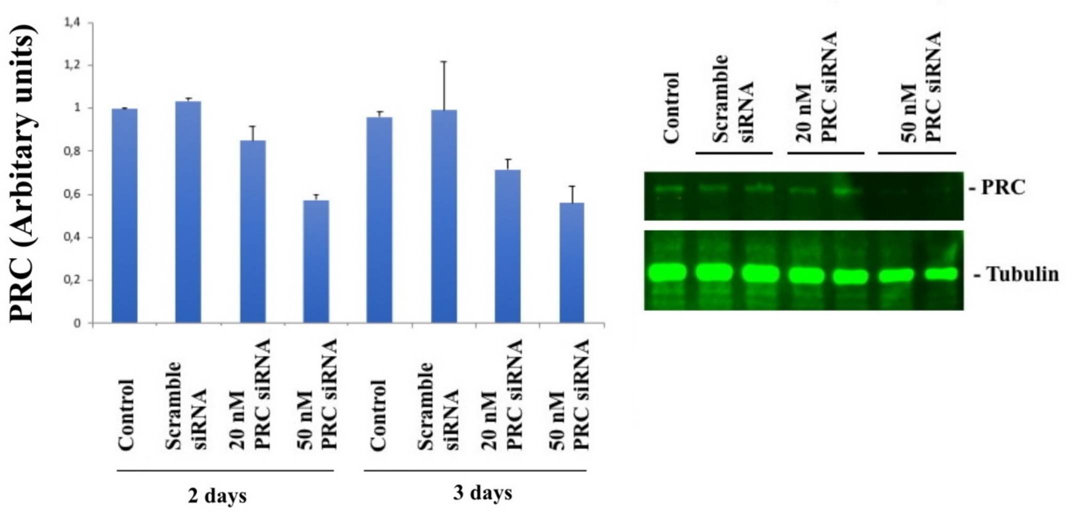

ESM Fig. 5. Knockdown of PRC in EndoC-βH1 cells. Cells were transfected with 50 nmol/L negative control (Sigma-Aldrich), 20 nmol/L or 50 nmol/L small interfering RNA (siRNA) (Ambion, life technologies) and Lipofectamine 2000 (Invitrogen) diluted in Opti-MEM (Invitrogen). After transfection for 3 h, DMEM/F12 medium was replaced and cultured for 48 or 72 hours before cell harvest. The expression levels of PRC and α-tubulin were measured by immunoblotting analysis and the results are representative for two independent experiments.
